# Supplementary figures and images for: Long-Term Kinetics of SARS-CoV-2 Neutralizing and Anti-Receptor Binding Domain Antibodies among Laboratory-Confirmed COVID-19 Cases in Delhi National Capital Region, India: A Prospective, One-Year Follow-Up Study
Source: J Clin Med. 2024 Jan 29;13(3):762. doi: 10.3390/jcm13030762 (PMC10856624; doi:10.3390/jcm13030762)

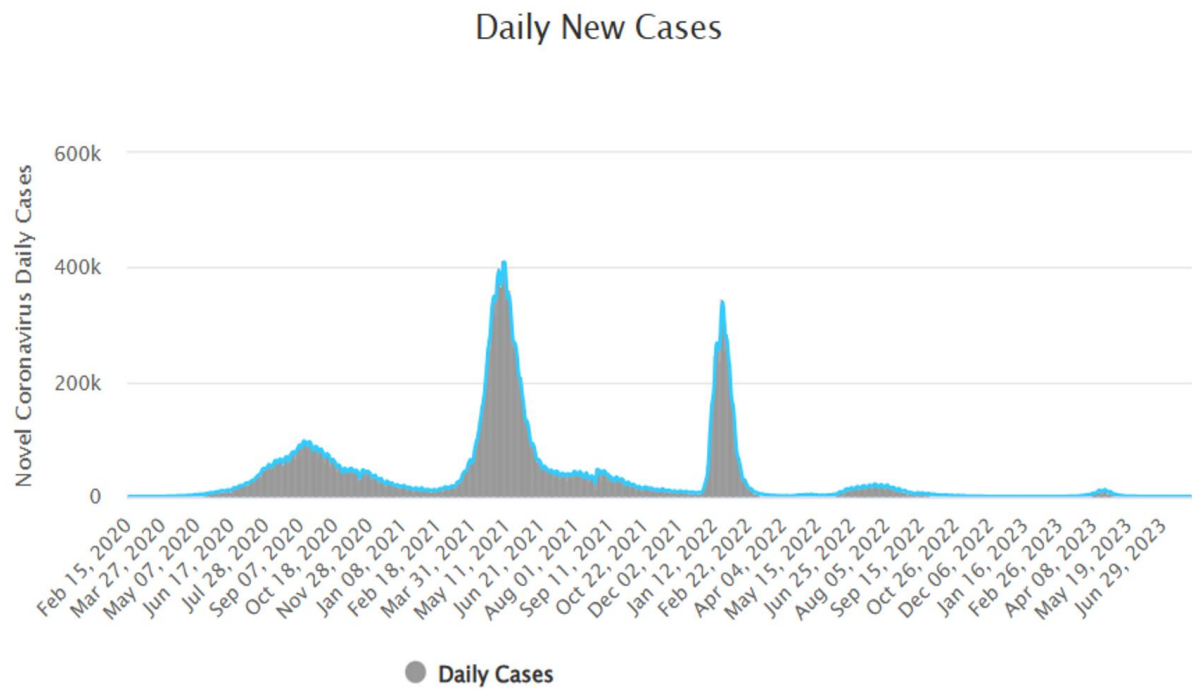

Figure S1 : Daily new cases of COVID-19 India [13,14].

Supplement: Supplementary file 1 [file jcm-13-00762-s001.zip › jcm-2780594-supplementary.pdf]
